# Cocaine-Induced Cardiomyopathy as a Rare Etiology of Severe Heart Failure in Young Adults: A Case Report

**DOI:** 10.7759/cureus.106598

**Published:** 2026-04-07

**Authors:** Pranali R Dave, Melkon Hacobian

**Affiliations:** 1 Medical School, California University of Science and Medicine, Colton, USA; 2 Cardiology, University of California Los Angeles David Geffen School of Medicine, Los Angeles, USA

**Keywords:** cocaine abuse, cocaine-induced cardiomyopathy, dilated cardiomyopathy (dcm), heart failure with reduced ejection fraction, reversible cardiomyopathy, stimulant cardiotoxicity, substance-related cardiac disease

## Abstract

Cocaine use is well known to cause acute cardiovascular events, but chronic cardiomyopathy is a less common consequence. We present a 27-year-old male with five years of cocaine use who developed acute decompensated heart failure, marked by severe biventricular dilation, an ejection fraction of 19%, pulmonary hypertension, and multiorgan congestion. After inpatient stabilization, he was started on guideline-directed medical therapy and abstained from cocaine. This case underscores that even profound cardiac dysfunction from cocaine can be substantially reversible with early recognition, aggressive therapy, and substance cessation.

## Introduction

Cocaine is one of the most widely used illicit stimulants, with an estimated 18 million users worldwide and approximately 5 million users in the United States [1]. Cardiovascular complications account for a large proportion of the morbidity and mortality associated with cocaine use, and emergency departments report over 60,000 annual visits for cocaine-related chest pain [2]. Acute intoxication is well known to provoke a range of cardiovascular events, including hypertension, tachyarrhythmias, coronary vasospasm, myocardial infarction, aortic dissection, and sudden cardiac death [3,4].

In addition to these acute events, chronic cocaine use has been increasingly recognized as a cause of progressive myocardial injury. Long-term exposure is associated with structural remodeling, left ventricular hypertrophy, myocardial fibrosis, and eventual systolic dysfunction [5,6]. Several studies have demonstrated that even asymptomatic users may develop subclinical cardiomyopathy, with measurable reductions in ejection fraction or diastolic impairment compared with non-users [7]. Among patients presenting with new-onset dilated cardiomyopathy, cocaine use should therefore be considered an important reversible etiology.

The pathophysiology of cocaine-induced heart failure is multifactorial. Key mechanisms include sympathetic overdrive, transient coronary vasoconstriction and thrombosis, direct myocardial sodium channel blockade leading to conduction delays and QRS widening, oxidative stress, and fibrosis and remodeling [2,4,6,7].

The prognosis of cocaine-induced cardiomyopathy is variable. Some patients progress to end-stage heart failure requiring advanced therapies, while others show significant recovery with strict abstinence and guideline-directed medical therapy [8,9]. Recognition of this entity is particularly important in young adults, who may otherwise lack traditional cardiovascular risk factors, and in whom early intervention may prevent long-term morbidity.

We report the case of a 27-year-old male with a history of heavy cocaine use who presented with acute decompensated heart failure and severe biventricular systolic dysfunction. This case highlights the clinical presentation, diagnostic evaluation, and management of cocaine-induced cardiomyopathy and underscores the potential for reversibility with abstinence and optimized medical therapy.

Despite increasing recognition of cocaine-related cardiovascular complications, severe biventricular failure with acute decompensation in young patients without traditional risk factors remains relatively uncommon. This case is particularly notable for the degree of cardiac dysfunction at presentation, including profound systolic impairment and pulmonary hypertension, as well as the rapid clinical improvement observed with guideline-directed therapy and substance cessation. Given the ongoing prevalence of cocaine use, this case highlights the importance of early recognition of substance-related cardiomyopathy as a potentially reversible cause of heart failure in young adults and underscores the need for heightened clinical suspicion in this population.

## Case presentation

A 27-year-old Korean male with a past medical history notable for chronic cocaine use over the past five years presented to the clinic with worsening shortness of breath for three weeks. He described exertional progressive dyspnea that had advanced to limiting daily activities, along with orthopnea and frequent episodes of paroxysmal nocturnal dyspnea. He also reported increasing bilateral lower extremity swelling, abdominal distention, and generalized fatigue. He denied chest pain, palpitations, fever, or recent upper respiratory infection. He denied recent cocaine use in the past month but acknowledged frequent use in the preceding years. His medical history was otherwise unremarkable except for a prior Epstein-Barr virus infection during his early twenties. He denied a family history of cardiomyopathy or sudden cardiac death.

On arrival, the patient appeared ill and in respiratory distress. Vital signs revealed a heart rate of 110 beats per minute, a blood pressure of 104/68 mmHg, a respiratory rate of 24 breaths per minute, an oxygen saturation of 95% on room air, and a temperature of 36.9°C. Jugular venous pressure was elevated to 10 cm above the sternal angle. Cardiac auscultation revealed a displaced apical impulse and an audible third heart sound (S3 gallop). A grade II/VI holosystolic murmur was heard at the apex, consistent with functional mitral regurgitation. Pulmonary examination demonstrated bibasilar rales. The abdomen was distended with shifting dullness, suggesting ascites. There was 2+ pitting edema in both lower extremities. Peripheral pulses were intact, and there were no stigmata of endocarditis or intravenous drug use.

Initial laboratory testing demonstrated renal and hepatic dysfunction. Laboratory findings are summarized in Table 1.

**Table 1:** Initial laboratory findings on presentation ALP, alkaline phosphatase; ALT, alanine aminotransferase; BNP, B-type natriuretic peptide; CBC, complete blood count.

Test	Value	Reference range
Creatinine	2.3 mg/dL	0.6-1.3 mg/dL
BNP	3000 pg/mL	<100 pg/mL
Sodium	132 mmol/L	135-145 mmol/L
ALP	160 U/L	44-147 U/L
ALT	60 U/L	7-56 U/L
Troponin	Normal	—
CBC	Normal	—

**Figure 1:**
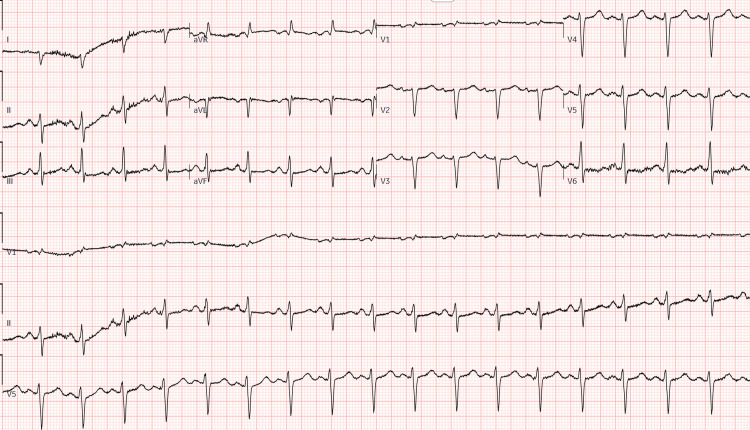
Twelve-lead electrocardiogram demonstrating sinus tachycardia with intraventricular conduction delay (QRS ~150 ms) without acute ischemic ST-segment elevation.

Twelve-lead electrocardiogram demonstrated sinus tachycardia at a rate of 110 beats per minute with a widened QRS duration of approximately 150 ms, consistent with an intraventricular conduction delay. No acute ischemic ST-segment elevations were observed (Figure 1).

**Figure 2:**
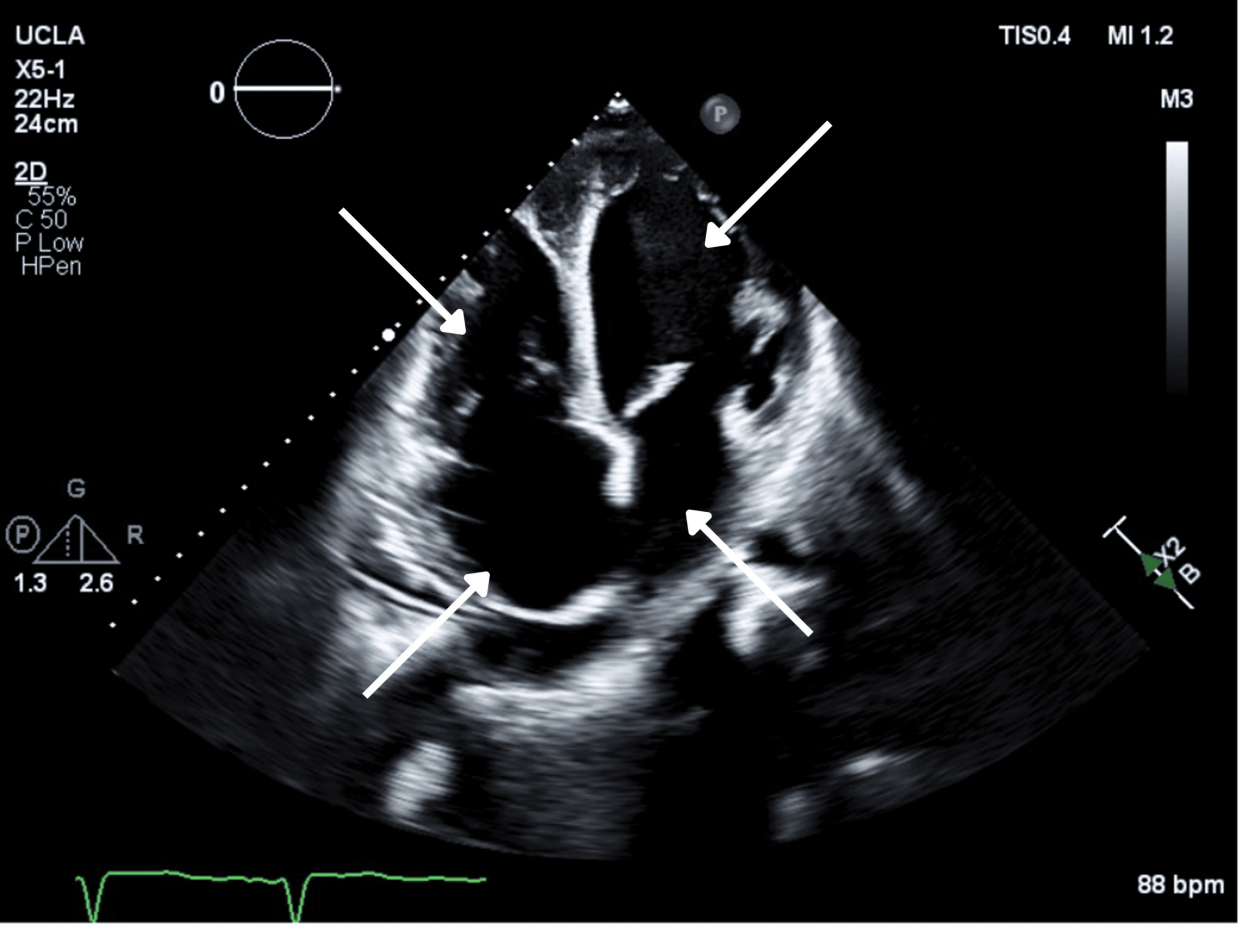
Transthoracic echocardiogram (apical four-chamber view) demonstrating biventricular dilation with biatrial enlargement (arrows).

**Figure 3:**
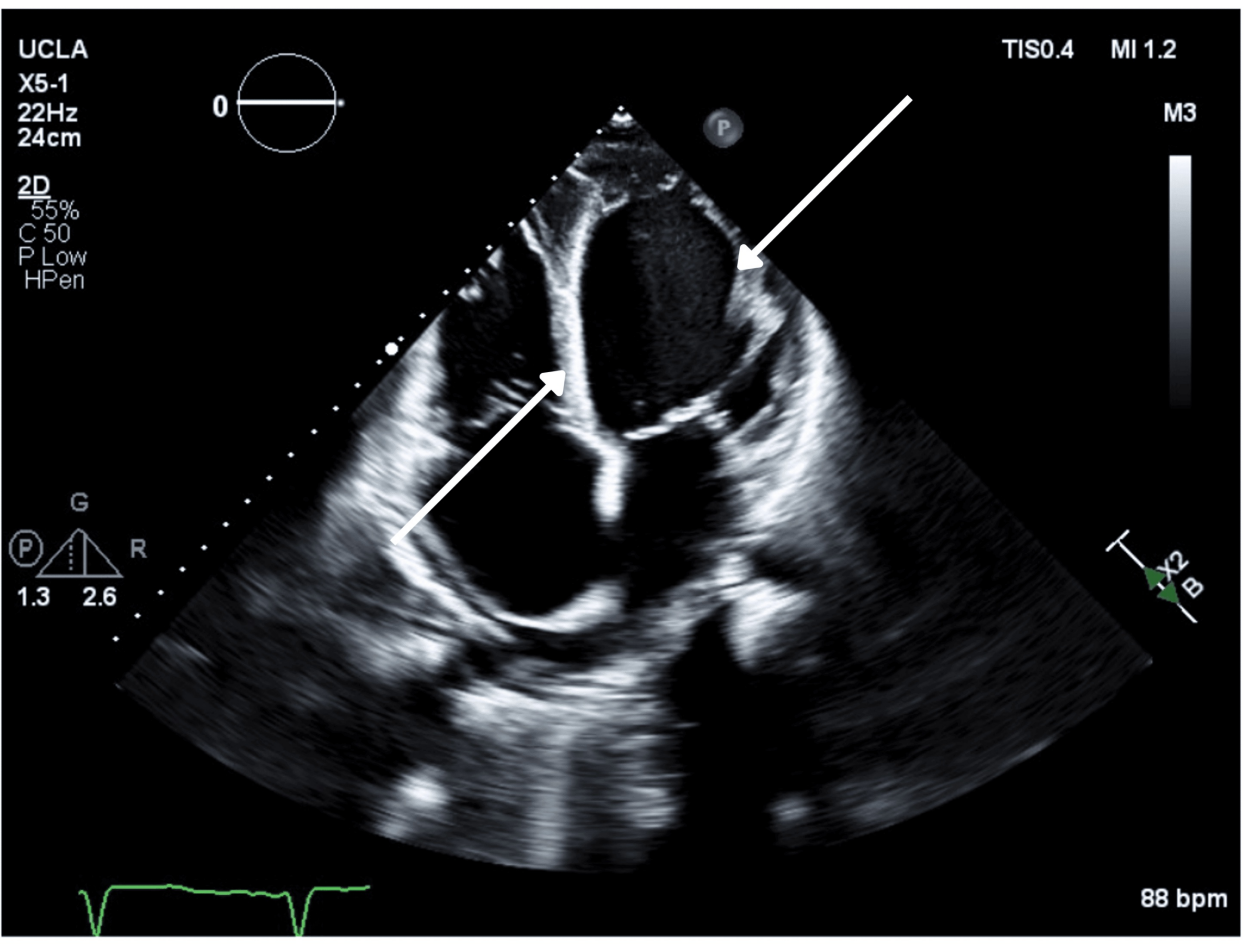
Transthoracic echocardiogram (apical four-chamber view) demonstrating severely reduced left ventricular systolic function with global hypokinesis (arrows).

Bedside transthoracic echocardiography revealed a severely dilated left ventricle with global hypokinesis and an estimated left ventricular ejection fraction of 19%. The right ventricle was also dilated with reduced systolic function. There was marked biatrial enlargement. Moderate central mitral regurgitation was present, consistent with annular dilatation. Estimated pulmonary artery systolic pressure was elevated at 65 mmHg, indicating severe pulmonary hypertension likely consistent with WHO Group 2 pulmonary hypertension secondary to left-sided heart dysfunction (Figures 2, 3) [10].

**Figure 4:**
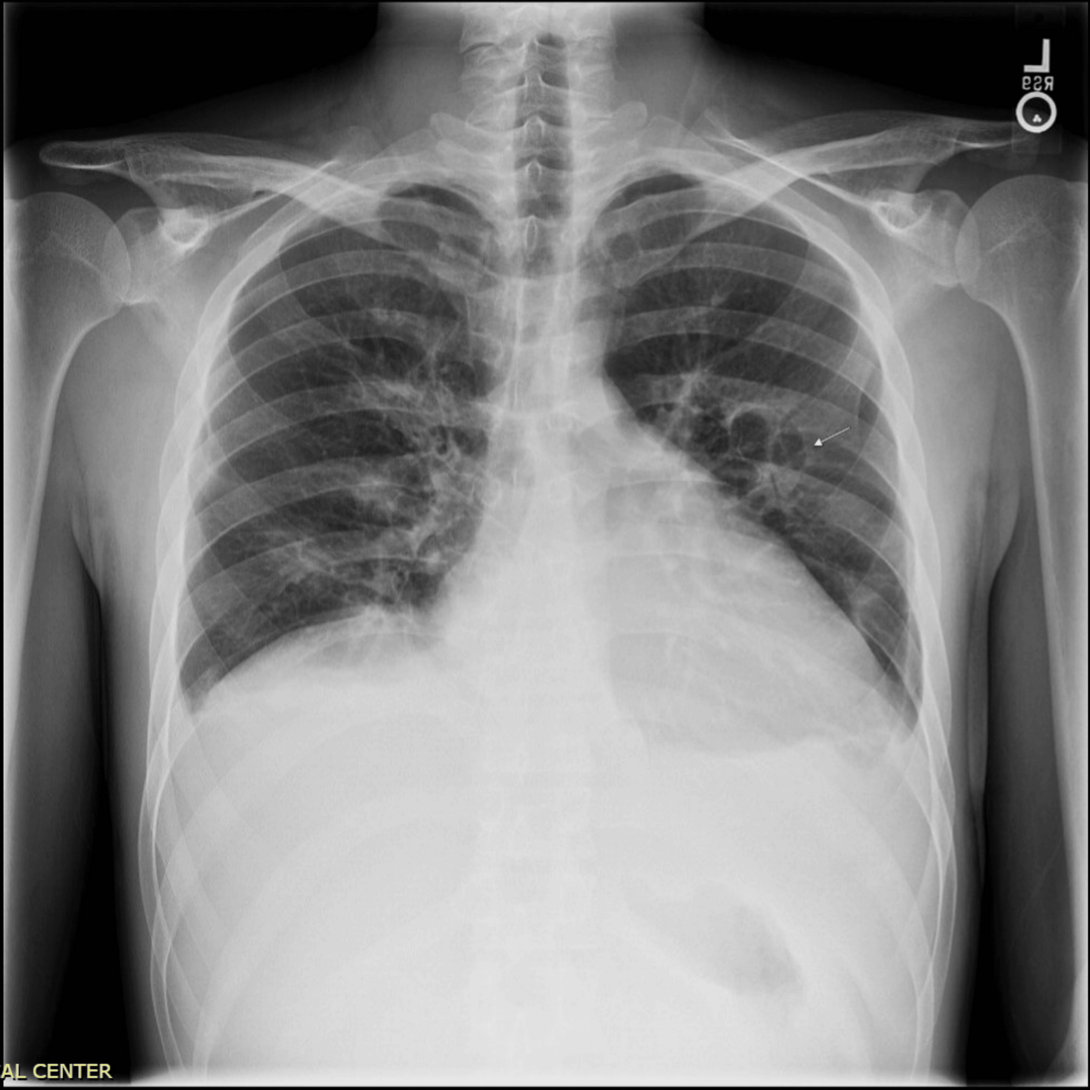
Chest radiograph demonstrating cardiomegaly with pulmonary venous congestion and bilateral interstitial opacities consistent with pulmonary edema.

Chest radiograph demonstrated cardiomegaly with a prominent cardiac silhouette and pulmonary venous congestion. There were diffuse interstitial markings in the lung bases consistent with pulmonary edema (Figure 4).

The patient was admitted with acute decompensated heart failure and initiated on intravenous diuresis with furosemide, resulting in adequate urine output and gradual improvement in dyspnea and peripheral edema. Following initial stabilization, GDMT for heart failure with reduced ejection fraction was initiated. This included sacubitril-valsartan 49/51 mg twice daily, empagliflozin 10 mg daily, and spironolactone 12.5 mg daily with close monitoring of renal function and electrolytes.

After hemodynamic stabilization, beta-blocker therapy was initiated with metoprolol succinate 25 mg daily. The patient was transitioned to oral furosemide 20 mg daily for maintenance diuresis. Digoxin 0.125 mg daily was initiated for additional rate control and symptomatic support.

The patient remained on continuous telemetry monitoring, and no sustained ventricular arrhythmias were observed during hospitalization. He received extensive counseling regarding cocaine cessation and was referred to addiction medicine services for outpatient follow-up.

## Discussion

Our patient’s presentation is striking for its severity and rapid decompensation in a young adult. Unlike cases where subclinical dysfunction is detected only via imaging or biomarker monitoring, this patient presented with overt congestive heart failure, markedly reduced ejection fraction (≈19%), biventricular dilation, pulmonary hypertension, and evidence of end-organ dysfunction, including acute kidney injury likely secondary to cardiorenal syndrome in the setting of volume overload and reduced cardiac output. This aligns with several published case reports showing that cocaine-induced cardiomyopathy, even when severe, may substantially improve if the patient achieves abstinence and receives optimal medical therapy [1,4,5]. For example, Cooper et al. described a patient who improved substantially after five months of cocaine cessation [1], and a recent case report demonstrated rapid recovery with GDMT and strict abstinence [8].

Management of cocaine-induced cardiomyopathy largely parallels non-ischemic dilated cardiomyopathy. Multiple reports show that sustained abstinence from cocaine use is perhaps the most important prognostic factor in achieving recovery of left ventricular function [1,5]. Use of angiotensin-converting enzyme (ACE) inhibitors (or angiotensin II receptor blockers/angiotensin receptor-neprilysin inhibitors), beta-blockers, mineralocorticoid receptor antagonists, and, where available, SGLT2 inhibitors is consistent with best practices. Beta-blocker use in particular has been controversial historically due to the theoretical risk of exacerbating vasoconstriction during acute stimulant exposure, but recent data suggest beta-blocker use may be beneficial when cocaine exposure is remote and the patient is stable [2,8].

In our case, the early introduction of an ACE inhibitor, an aldosterone antagonist, cautious beta-blockade only after stabilization, and rigorous diuresis have contributed to rapid clinical improvement and a marked recovery of ejection fraction. With the continued prevalence of stimulant use, clinicians must maintain a high index of suspicion for cocaine-induced cardiomyopathy as an underrecognized but potentially reversible cause of severe heart failure.

## Conclusions

This case illustrates a rare but clinically important manifestation of chronic cocaine use: severe, biventricular dilated cardiomyopathy with acute decompensated heart failure in a young adult without traditional cardiovascular risk factors. The combination of markedly reduced ejection fraction, pulmonary hypertension, and multiorgan congestion at presentation underscores the profound systemic impact that cocaine cardiotoxicity can have. Importantly, the patient’s dramatic improvement in ventricular function with abstinence and timely initiation of guideline-directed therapy demonstrates that even severe cocaine-induced cardiomyopathy can be reversible.

For clinicians, this case highlights the need to consider substance use in the differential diagnosis of new-onset cardiomyopathy, particularly in young patients, and to recognize that recovery potential may be substantial if the etiology is promptly identified. Early detection, comprehensive management, and strong emphasis on cocaine cessation are crucial for optimizing outcomes in this unique patient population.

## References

[1] Cooper CJ, Said S, Alkhateeb H (2013). Dilated cardiomyopathy secondary to chronic cocaine abuse: a case report. BMC Res Notes.

[2] Arenas DJ, Beltran S, Zhou S, Goldberg LR (2020). Cocaine, cardiomyopathy, and heart failure: a systematic review and meta-analysis. Sci Rep.

[3] Havakuk O, Rezkalla SH, Kloner RA (2017). The cardiovascular effects of cocaine. J Am Coll Cardiol.

[4] Afonso L, Mohammad T, Thatai D (2007). Crack whips the heart: a review of the cardiovascular toxicity of cocaine. Am J Cardiol.

[5] Bertolet BD, Freund G, Martin CA, Perchalski DL, Williams CM, Pepine CJ (1990). Unrecognized left ventricular dysfunction in an apparently healthy cocaine abuse population. Clin Cardiol.

[6] Liaudet L, Calderari B, Pacher P (2014). Pathophysiological mechanisms of catecholamine and cocaine-mediated cardiotoxicity. Heart Fail Rev.

[7] Abraham JR, Butany J, Leong S (2009). Cocaine cardiotoxicity: a review of the pathophysiology, pathology, and treatment options. Am J Cardiovasc Drugs.

[8] Kamel I, Salah A, Esteghamati S, Dietzuis H (2023). Rapid recovery from cocaine-induced cardiomyopathy: a case report. Cureus.

[9] Chokshi SK, Moore R, Pandian NG, Isner JM (1989). Reversible cardiomyopathy associated with cocaine intoxication. Ann Intern Med.

[10] Ratwatte S, Celermajer DS (2024). The latest definition and classification of pulmonary hypertension. Int J Cardiol Congenit Heart Dis.

